# Diagnostic performance of a novel point-of-care test for the diagnosis of visceral leishmaniasis in Sudan: A Comparative Accuracy Study

**DOI:** 10.1371/journal.pntd.0012905

**Published:** 2025-04-08

**Authors:** Elfadil Abass, Durria Mansour, Zaki Abdalla, Hassan Altaher, Mootaz Sliman, Hussam Osman, Rabie Ali Babiker, Mohamed Salah, Elmohaned Omer, Elwaleed Elamin, Alexander Visekruna, Ulrich Steinhoff, Rouzbeh Mahdavi

**Affiliations:** 1 Department of Clinical Laboratory Science, College of Applied Medical Sciences, Imam Abdulrahman Bin Faisal University, Dammam, Saudi Arabia; 2 Ahfad Biomedical Research Laboratory (ABRL), School of Health Sciences, Ahfad University for Women, Omdurman, Sudan; 3 Laboratory and Blood Bank Administration, Ministry of Health, Gedaref, Sudan; 4 Mahmoud Abdalla Idris Medical Compound, Gedaref Teaching Hospital, Ministry of Health, Gedaref, Sudan; 5 Faculty of Medical and Health Sciences, Liwa College, Abu Dhabi, United Arab Emirates; 6 Department of Medical Microbiology, Faculty of medicine and Health Sciences, University of Gedaref, Gedaref, Sudan; 7 Department of Medical Microbiology, Faculty of Medicine, Kassala University, Kassala, Sudan; 8 Department of Medical Microbiology, Faculty of Medical Laboratory Sciences, Alzaeem Alazhari University, Khartoum, Sudan; 9 Institute of Medical Microbiology and Hospital Hygiene, Philipps University of Marburg, Marburg, Germany; U.S. Food and Drug Administration and Center for Biologics Evaluation and Research, UNITED STATES OF AMERICA

## Abstract

East-African countries suffer from a high incidence of Visceral Leishmaniasis (VL). Despite the availability of several diagnostic tests for VL, diagnosis remains a challenge in these countries. Due to the inefficiency of current diagnostics, a novel rapid diagnostic test (RDT) (INgezim Leishma-CROM) based on a recombinant multi-epitope kinesin antigen, rKLi8.3, was compared with the currently used rK39 RDT (IT-LEISH) in Eastern Sudan.

**Methods:**

This study enrolled 107 VL suspects who attended a health centre in Gedaref between October 2022 and June 2023. Diagnostic accuracy was assessed by comparing the performance of the new index test (INgezimLeishma-CROM) with the comparator test (IT-LEISH) and parasitological examination as reference standard.

**Results:**

Of 107 VL suspects screened by parasitological examination, 77 VL patients were smear positive and 30 were smear negative. Independent serological testing of these patients using INgezim Leishma-CROM showed a sensitivity of 98.7% [95% CI 92.23-99.97] and specificity of 92.5% [95% CI 75.71-99.09]. For IT-LEISH, both sensitivity [95% CI 84.39-97.20] and specificity [95% CI 75.71-99.09] were 92.5%. INgezim Leishma-CROM demonstrated increased diagnostic accuracy (97.2%) compared to IT-LEISH RDT (92.5%). Both RDTs gave positive results in 2 cases from the smear negative group that were previously treated for VL. All other non-VL cases (malaria, typhoid, brucellosis) were negative in both RDTs, showing 100% specificity, while VL patients co-infected with malaria were positive in both tests. Within the smear-negative group, 3 VL symptomatic cases that had been previously treated but still show clinical signs were all positive with INgezim Leishma-CROM but only 2 cases were positive with IT-LEISH.

## Author summary

Visceral Leishmaniasis (VL), also known as Kala-azar, is a neglected tropical disease prevalent in regions such as Sudan, India, Bangladesh, and East Africa. In Sudan, VL is transmitted by the sandfly *Phlebotomus orientalis*, with rural areas like Gedaref and White-Nile State facing the highest burden. Despite being treatable, VL causes mortality, particularly in rural areas, due to limited access to effective diagnostics and treatment. Current diagnostic methods, including microscopic parasite detection and rK39-based rapid diagnostic tests (RDTs), are hindered by low sensitivity and are not ideal for resource-poor regions.

In response to this, a new recombinant kinesin antigen (rKLi8.3) from a Sudanese *L. infantum* strain has been developed to improve VL diagnosis. A prospective study was conducted in Gedaref, Eastern Sudan, to evaluate the diagnostic accuracy of this new antigen by comparing two diagnostic tests: INgezim Leishma-CROM and IT-LEISH.

In conclusion, both a previous retrospective and the current prospective study demonstrate that INgezim Leishma-CROM is suitable for field diagnosis and shows enhanced diagnostic performance in East Africa. This test offers a significantly improved alternative to the currently used RDTs in VL endemic areas.

## Introduction

Visceral Leishmaniasis (VL) or Kala-azar is the second most lethal, neglected tropical disease (NTD) endemic in India, Bangladesh, East-Africa, in Mediterranean countries and Brazil [1,2]. In Sudan VL is transmitted to humans and animals through the bite of sandfly *Phlebotomus orientalis* [3,4].

Sudan is one of the VL endemic countries in East Africa with the highest VL burden in the world, with 2000-7000 new infections per year, contributing 57% of the global annual cases (50,000-90,000) [5]. Most of these cases were reported in rural areas in Gedaref and White-Nile State. Even though, VL is treatable and curable, 52 and 46 people died from the disease in Sudan in 2019 and 2020, respectively [6].

Poverty, malnutrition and immunodeficiency are known risks for developing VL, and migration due to food shortages, civil unrest and war has led to a high incidence and prevalence of VL in this region [7,8].

Therefore, early and accurate diagnosis and treatment are critically important to control and manage VL in East-Africa. At rural VL-endemic areas in Sudan, diagnosis of this disease is primarily based on microscopic detection of amastigote *Leishmania* or rK39 based RDTs. Both methods are applicable at primary health care centres and rural settings. Microscopic detection of parasites remains the reference standard for VL diagnosis because of its high specificity. However, the sensitivity is generally low in East Africa and depends on the parasitemia and skills of the person that carries out the work. Aspiration of infected tissues such as lymph nodes, spleen or bone marrow is invasive and bears the risk of infection and other complications. It is increasingly recognized that in East Africa, both microscopy and rK39-based RDTs are not appropriate due to low sensitivity and variable specificity [9]. Therefore, new reliable and non-invasive diagnostic methods are urgently needed in resources-poor countries [10]. Accordingly, the WHO has prioritized the development of better RDTs for VL in East Africa in the 2021-2030 roadmap for NTDs [11].

Recently, a new recombinant kinesin antigen (rKLi8.3) from a Sudanese *L. infantum strain* (MHOM/SD/82/GILANI) was developed to improve VL serodiagnosis in East Africa and evaluated in a retrospective study with sera from Sudan and other endemic countries [12]. Here, a prospective diagnostic accuracy study was performed to diagnose VL suspects at a health centre in the VL endemic area of Gedaref, Eastern Sudan, by comparing INgezim Leishma-CROM with IT-LEISH.

## Methods

### Ethics statement

The study was approved by the University Ethical Review Committee (UERC0992) of Ahfad University for Women, Omdurman, Sudan. Written informed consent was obtained from all participants prior to enrolment in the study and the STARD guidelines for reporting diagnostic accuracy studies were followed. For underage patients, verbal consent was obtained from parents/or guardians.

### Study design and participants

This VL diagnostic study was designed as a comparative accuracy study, comparing the sensitivity and specificity of the new INgezimLeishma-CROM RDT (GSD Madrid) with the currently available rK39 RDT (IT-LEISH, Biorad) in Sudan. The study was performed without bias towards non-inferiority, equivalence, or superiority to objectively evaluate the performance of both RDTs. Patients from Gedaref and rural areas with suspected VL and those with a history of VL who were admitted to the Gedaref Health Centre in Eastern Sudan were enrolled between 23 October 2022 and 3 June 2023. Patients with fever of 2 weeks’ duration and spleno-hepatomegaly and/or weight loss, lymphadenopathy or other VL-supportive symptoms (Table 1) were considered as VL suspects according to national and international guidelines [2]. The STARD guidelines for reporting diagnostic accuracy studies were followed [13]. 107 patients were tested with the currently used and recommended rK39 RDT (IT-LEISH, Biorad), the new rKLi8.3 RDT (INgezimLeishma-CROM, GSD Madrid) and by parasitological examination as reference standard [14]. Due to the recent armed conflict in Sudan, which started on 15 April 2023, the study had to be terminated with a total of 107 participants.

**Table 1:** Demographic and clinical characteristics of VL suspects.

Characteristics	Cases (n=107)
Age/year*	
Median	15
Mean±SD	22±17.4
Range	1-75
0-14	48 (49%)
15-29	21 (21.4%)
30-44	19 (19.4%)
45-59	6 (6.1%)
≥60	4 (4.1%)
Sex	
Male	66 (61.7%)
Female	41 (38.3%)
Medical history	
Cured VL**	2 (1.8%)
VL relaps***	3 (2.8%)
Previous CL	0 (0%)
HIV history	0 (0%)
Symptoms	
Fever	107 (100%)
Abdominal pain/distention/mass	47 (43.9%)
Vomiting	53 (49.5%)
Loss of appetite	102 (95.3%)
Weight loss	95 (88.8%)
Bleeding (Epistaxis)	1 (0.9%)
Intermittent diarrhea	37 (34.6%)
Coughing	23 (21.5%)
Joint pain	106 (99.1%)
Signs	
Emaciation	70 (65.4%)
Pallor	10 (9.3%)
Skin darkness	10 (9.3%)
Other skin change	3 (2.8%)
Lymphadenopathy	78 (72.9%)
Splenomegaly	72 (67.3%)
Hepatomegaly	67 (62.6%)
Ascites	5 (4.7%)
Oedema	0 (0.0%)
Other Infections	
Malaria	13 (12.1%)
Typhoid	9 (8.4%)
Brucellosis	3 (2.8%)
Coinfections	
VL/Malaria	8 (7.47%)

### Procedures

Demographic and clinical data were obtained from all participants using a standard data collection form and results were recorded in code. Laboratory diagnosis of VL was performed by direct detection of amastigotes from inguinal lymph node aspirates (LNA). Lymph node smears were fixed with methanol, stained with Giemsa, and examined by light microscopy at 1000x magnification. Approximately 100 microscopic fields were examined by 2 experienced technicians counting extracellular or intracellular amastigote forms in monocytes and macrophages. Parasite density was reported ranging from 0 (no parasites) to +6 (parasites greater than 100 per field) [15]. Patients with typical clinical signs and symptoms of VL in combination with either positive parasitology or serology were considered confirmed VL. Based on smear results, patients were divided into smear positive (n=77) and smear negative (n=30) cohorts. The latter group also included patients with malaria (n=13), typhoid fever (n=9), and brucellosis (n=3). INgezim Leishma-CROM and IT-LEISH RDTs were used in a head-to-head comparison on fresh peripheral blood (10-20µl) from fingerprick of all patients and used immediately for sero-diagnosis. Results were read independently by 2 examiners indicating the validity of the RDTs by the appearance of the control band. Invalid tests were repeated.

Diagnosis of malaria was based on microscopic examination of Giemsa-stained peripheral blood smears. Diagnosis of typhoid fever and brucellosis was done by semi-quantitative determination of specific antibodies using standard serological tests (Widal test & Brucel-A/M, BioMed Diagnostics, Cairo, Egypt).

### Statistical analysis

Diagnostic accuracy was assessed by comparing the sensitivity and specificity of the new index test (INgezimLeishma- CROM) with the comparator test (IT-LEISH) and parasitological examination as reference standard. The performance of each RDT was evaluated in terms of sensitivity, specificity, positive predictive value (PPV), negative predictive value (NPV) and accuracy (AC). Index values were estimated at 95% confidence intervals (95% CI) and MedCalc statistical software was used to calculate performance indexes. Demographic and clinical data were presented in absolute numbers and percentage of the whole study group. The degree of agreement between both RDTs with the parasitological examination was determined using Kappa index (κ) values with 95% confidence intervals. Kappa index values were interpreted according to Fleiss et al.: 0.00–0.20, slight agreement; 0.21–0.40, fair agreement; 0.41–0.60, moderate agreement; 0.61–0.80, substantial agreement; 0.81–1.00, almost perfect agreement [16].

## Results

**Fig 1:**
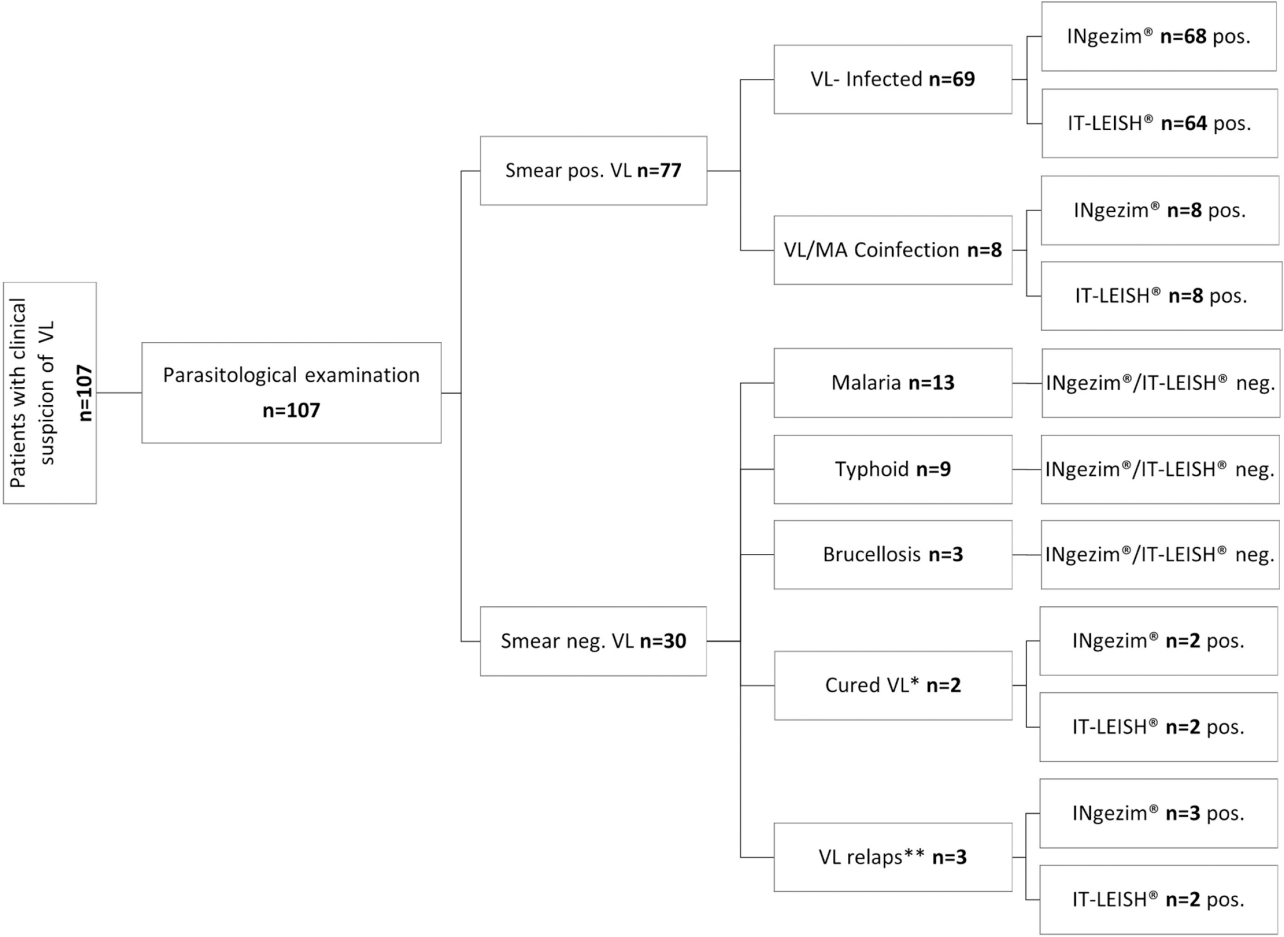
Diagnostic assessment and patient flow of VL suspects in Gedaref, Sudan.

107 VL suspects were included in a diagnostic accuracy study at a health care centre in Gedaref. The mean age of VL suspects was 22 ± 17.4 years (median, 15); 66 persons were male (61.7%) and 41 females (38.3%). Major clinical signs and symptoms were fever (100%), joint pain (99.1%), loss of appetite (95.3%), weight loss (88.8%), lymphadenopathy (72.9%), splenomegaly (67.3%), and hepatomegaly (62.6%) (Table 1). Following parasitological examination, VL suspects were divided into 77 smear positive and 30 smear negative cases, (Fig 1). The latter group included infections other than VL (malaria, typhoid fever, brucellosis) and 2 individuals without typical clinical signs of VL (cured VL) who had been treated with AmBisome and 3 symptomatic VL (VL relaps) who despite treatment with AmBisome showed typical clinical signs at the time of the study. 8 VL suspects were co-infected with malaria, none of the patients had a history of cutaneous Leishmaniasis (CL) or HIV infection. Independent of the parasitological outcome, INgezim Leishma-CROM was compared head-to-head with IT-LEISH for serodiagnosis of all VL suspects.

Sensitivity was determined using 69 VL, 8 VL-Malaria co-infected and 3 VL relaps cases and specificity was calculated on 25 non-VL cases (13 malaria, 9 typhoid, 3 brucellosis) and 2 cured VL cases. Accordingly, INgezim Leishma-CROM showed a sensitivity of 98.75% (95% CI 93.23-99.97%) and specificity of 92.59% (95% CI 75.71-99.09%) and IT-LEISH achieved a sensitivity of 92.5% (95% CI 84.39-97.2%) and specificity of 92.59% (95% CI 75.71-99.09%) (Table 2). Within the 77 smear positive group which included 8 VL-Malaria co-infected patients, INgezim Leishma-CROM and IT-LEISH detected 76 and 72 VL patients, respectively. While all VL-Malaria co-infections were detected by both RDTs, no cross-reactivity was observed within the 25 patients suffering from Malaria, Typhoid and Brucellosis. Within the smear negative group, 5 well documented patients previously received VL treatment. Two patients showed clinical improvement after treatment and were considered cured but showed positive results in both RDTs. Three patients showed typical VL clinical signs but were smear negative, all of them were positive with INgezim Leishma-CROM and 2 of them with IT-LEISH.

**Table 2:** Diagnostic performance of INgezim Leishma-CROM and IT-LEISH.

RDTs	TP	FN	TN	FP	Performance Index (%) at 95% CI					
					Sensitivity (%)	Specificity (%)	PPV (%)	NPV (%)	κ	AC (%)
INgezim	79	1	25	2	98.75 (93.23 to 99.97)	92.59 (75.71 to 99.09)	97.53 (91.23 to 99.34)	96.15 (78.04 to 99.43)	0.925 (84.1 to 100)	97.2 (92.02 to 99.42)
IT-LEISH	74	6	25	2	92.50 (84.39 to 97.20)	92.59 (75.71 to 99.09)	97.37 (90.69 to 99.29)	80.65 (65.71 to 90.06)	0.801 (68.6 to 93.6)	92.52 (85.80 to 96.72)

Abbreviations: (RDT) rapid diagnostic test; (TP) true positive; (FN) false negative; (TN) true negative; (FP) false positive; (CI) confidence interval; (PPV) positive predictive value; (NPV) negative predictive value; (k) Kappa index; (AC) accuracy. Sensitivity was determined using 77 smear positive VL cases and 3 VL relaped patients. Specificity was estimated using 25 non-VL cases including 13 malaria, 9 typhoid, 3 brucellosis and 2 with cured VL cases.

The PPV, NPV and AC of INgezim Leishma-CROM were 97.53% (91.23% to 99.34%), 96.15% (78.04% to 99.43%) and 97.20% (92.02% to 99.42%). For IT-LEISH the PPV value was 97.37% (90.69% to 99.29%), NPV 80.65% (65.71% to 90.06%) and AC 92.52% (85.80% to 96.72%).

The Kappa index (κ) values (95% CI) for IT-LEISH was 0.801 and for INgezim Leishma-CROM 0.925.

### Handling and Reading of RDTs

INgezim Leishma-CROM showed valid results in all 107 cases, as evidenced by clear test (T) and control (C) lines. Using the IT-LEISH, 5 cases (4.7%) showed unclear test and/or control bands and test had to be repeated.

VL, visceral leishmaniasis; CL, cutaneous leishmaniasis; HIV, human immunodeficiency virus. ^*^ Data available for 98 patients. ^**^ Patients were previously treated with Pentostam and continued treatment with AmBisome at the time of the study and showed no typical VL clinical signs. ^***^ Patients were treated with AmBisome and continued to show clinical signs of VL.

## Discussion

Although several RDTs for VL exist, IT LEISH and Kalazar Detect are most commonly used in Sudan. Both rely on the rK39 kinesin antigen, showing a varying sensitivity by region. Despite the high sensitivity in India (98.8%) and Brazil (92%), IT LEISH has lower sensitivity in East Africa (87.2%) due to regional polymorphisms of the rK39 antigen [17–19]. To address this, the rKLi8.3 antigen, based on a conserved kinesin sequence from a Sudanese *L. infantum* strain, was developed and showed superior diagnostic performance compared to IT LEISH in a retrospective study in Sudan [12]. Here, INgezim Leishma-CROM and IT-LEISH were compared in prospective accuracy study in Gedaref, Sudan.

Among smear-positive patients, INgezim Leishma-CROM had a sensitivity of 98.7%, while IT-LEISH had 92.5%. Both tests also correctly identified 8 VL patients co-infected with malaria. In smear-negative patients, none of the RDTs gave false positives for malaria, typhoid, or brucellosis, though two VL patients with past AmBisome treatment were false positive, likely due to persistent antibodies [20–22].

Three symptomatic but smear-negative patients that have been treated with AmBisome were tested positive with INgezim Leishma-CROM and two with IT-LEISH. They were classified as VL true positives. Presence of VL-specific antibodies together with typical clinical signs after treatment indicates ongoing infection, due to incomplete elimination of the parasite or relapse of VL, particularly in immunocompromised individuals [23,24].

Test accuracy depends on the composition of the population and the reliability of the reference test, which may vary in sensitivity (52-65%) for LNA in Sudan [25–28]. Molecular diagnosis, while ideal, is not feasible in East Africa [29].

In Gedaref, INgezim Leishma-CROM demonstrated a sensitivity of 98.75% and a specificity of 92.59%, meeting WHO guidelines for the diagnosis of VL with a sensitivity of greater than 95% [9].

Although VL/HIV co-infection complicates diagnosis, war conditions and its low prevalence in eastern Sudan, did not allow testing for HIV co-infection [30,31].

A common issue for all serological tests is the persistence of specific antibodies up to several years. Therefore antibody-based tests for VL diagnosis must always be used in combination with a standardized clinical definition or other diagnostic methods [22,29].

The limitations of our study was the restricted sample size, which was constrained by the onset of war in Sudan. With only 107 patients enrolled, the power of our study to detect smaller differences in diagnostic performance may be limited. Despite this, the study provides valuable insights into the performance of the novel POC test for VL in Sudan. Further studies with larger sample size are recommended to validate our findings.

In conclusion, a previous retrospective - and the current prospective study demonstrate that INgezim Leishma-CROM is suitable for field diagnosis and show enhanced diagnostic performance in East Africa, providing an improved alternative to the currently used RDTs in this endemic area.
